# Weighted ASTRID: fast and accurate species trees from weighted internode distances

**DOI:** 10.1186/s13015-023-00230-6

**Published:** 2023-07-19

**Authors:** Baqiao Liu, Tandy Warnow

**Affiliations:** Department of Computer Science, University of Illinois Urbana-Champaign, Urbana, IL USA

**Keywords:** Species tree estimation, ASTRID, ASTRAL, Multi-species coalescent, Incomplete lineage sorting

## Abstract

**Background:**

Species tree estimation is a basic step in many biological research projects, but is complicated by the fact that gene trees can differ from the species tree due to processes such as incomplete lineage sorting (ILS), gene duplication and loss (GDL), and horizontal gene transfer (HGT), which can cause different regions within the genome to have different evolutionary histories (i.e., “gene tree heterogeneity”). One approach to estimating species trees in the presence of gene tree heterogeneity resulting from ILS operates by computing trees on each genomic region (i.e., computing “gene trees”) and then using these gene trees to define a matrix of average internode distances, where the internode distance in a tree *T* between two species *x* and *y* is the number of nodes in *T* between the leaves corresponding to *x* and *y*. Given such a matrix, a tree can then be computed using methods such as neighbor joining. Methods such as ASTRID and NJst (which use this basic approach) are provably statistically consistent, very fast (low degree polynomial time) and have had high accuracy under many conditions that makes them competitive with other popular species tree estimation methods. In this study, inspired by the very recent work of weighted ASTRAL, we present weighted ASTRID, a variant of ASTRID that takes the branch uncertainty on the gene trees into account in the internode distance.

**Results:**

Our experimental study evaluating weighted ASTRID typically shows improvements in accuracy compared to the original (unweighted) ASTRID, and shows competitive accuracy against weighted ASTRAL, the state of the art. Our re-implementation of ASTRID also improves the runtime, with marked improvements on large datasets.

**Conclusions:**

Weighted ASTRID is a new and very fast method for species tree estimation that typically improves upon ASTRID and has comparable accuracy to weighted ASTRAL, while remaining much faster. Weighted ASTRID is available at https://github.com/RuneBlaze/internode.

## Introduction

Species tree estimation is a common task in phylogenomics and is a prior step in many downstream analyses (e.g., estimating divergence, understanding adaptation). Despite the recent increase in the availability of genome-scale data, species tree estimation remains challenging due to gene tree heterogeneity, where gene trees (the evolutionary history of genes) differ from species trees [1]. Among common factors for gene tree heterogeneity, incomplete lineage sorting (ILS), a population-level process modeled statistically by the multi-species coalescent (MSC) [2, 3], is extremely common and well-studied.

A standard approach to species-tree reconstruction under the presence of ILS is to concatenate the alignments of the individual genes and running a maximum likelihood (ML) heuristic on the combined alignment. This simple approach, however, has been established to be statistically inconsistent under the MSC, and can even be positively misleading, returning a wrong topology with probability converging to 1 as the number of genes increases [4, 5]. Empirically, concatenation can also suffer from degraded accuracy under higher levels of ILS, and can be affected by scalability issues under large data [6, 7]. In response, many accurate ILS-aware methods have since been developed. Those that are most commonly used in practice fall into a class of so-called “summary methods”, where gene trees are first independently estimated from each genomic region, and the inferred gene trees are then used as input to the summary method, which outputs an estimated species tree (i.e., that summarizes the information in the input gene trees).

In recent years, many summary methods that are statistically consistent under the MSC have been developed, such as MP-EST [8], NJst [9], ASTRAL [6], ASTRID [10], FASTRAL [11], and wQFM [12]. Many of these methods are scalable to thousands of species with genomic-scale data (i.e., with thousands of genes). Among these methods, ASTRAL is the most well known, and has shown improved accuracy in comparison to many other methods in many simulation studies [13, 14]. Moreover, when the gene trees are adequately accurate and ILS is sufficiently high, ASTRAL tends to be more accurate than concatenation [7]. However, ASTRAL and summary methods more generally are not statistically consistent when given estimated gene trees and can be positively misleading in some model conditions [5]. Moreover, it is well known that accuracy for summary methods degrades as gene tree error increases [7, 15], and when gene tree error is sufficiently high, better accuracy may be obtained from concatenation, even under very high ILS, and even in the anomaly zone [7].

Alternative approaches to summary methods have also been developed that can be robust to conditions that lead to gene tree estimation error (e.g., gene alignments that have low phylogenetic signal due to being too short or evolving too slowly). Examples of such methods include methods that statistically co-estimate the gene trees and species trees, such as StarBeast [16] or StarBeast2 [17]. However, these co-estimation methods are very computationally intensive, as runtime is impacted by the number of genes as well as the number of species, so that StarBeast is limited to datasets that have perhaps 20 or 30 species and at most 100 genes [18]. Some other methods operate without ever estimating gene trees, including SVDquartets [19] and LilyQ [20]; these operate by computing quartet trees from the alignment and then combine the quartet trees into a tree on the full set of species using quartet amalgamation methods. Of these, LilyQ has perhaps the best accuracy and can be more accurate than ASTRAL when gene tree estimation error is high. However, these quartet-based methods are computationally intensive if all quartet trees must be computed, and subsampling quartet trees rather than computing and aggregating all $$\left( {\begin{array}{c}n\\ 4\end{array}}\right)$$ quartet trees is expected to reduce accuracy (see discussion in [21]). Thus, large-scale species tree estimation in the presence of ILS has substantial computational challenges, as surveyed in [22].

This sensitivity of summary methods to gene tree error has motivated approaches preprocessing the gene trees to improve the quality of the signal. Although throwing out inaccurate gene trees generally does not help [7], statistical binning [23, 24] and contracting low-support branches [14] improved accuracy for summary methods on many conditions. Nonetheless, these approaches require setting arbitrary thresholds: statistical binning requires a threshold to determine which branches are trustworthy, and contracting low-support branches also requires such a threshold. Suboptimal parameter selection in either case can lead to little accuracy improvement, or even worse, degraded accuracy compared to simply running on the original input [14, 24]. Thus, pragmatically, accurately applying such methods faces the difficulty of parameter selection.

Very recently, Zhang and Mirarab introduced weighted ASTRAL [25]. By directly incorporating gene tree uncertainty into the ASTRAL optimization problem, weighted ASTRAL improved ASTRAL in accuracy under all of their tested conditions. Notably, under conditions where concatenation proved more accurate than unweighted ASTRAL, weighted ASTRAL achieved the largest improvement, shrinking substantially the long known gap [13, 26, 27] between summary methods and concatenation under low gene signal. More specifically, (unweighted) ASTRAL heuristically searches for a species tree that maximizes the amount of quartet trees (unrooted four-taxon tree) shared with the input gene trees. By using branch support and lengths to weigh the reliability of gene tree quartets, weighted ASTRAL instead heuristically maximizes the weighted agreement with respect to the input gene trees, effectively discounting the contribution of unreliable quartets. Weighted ASTRAL is threshold-free, was shown to be more accurate than running ASTRAL on contracted gene trees [25], and in fact might be the most accurate summary method under ILS that can scale to large datasets.

Here, inspired by weighted ASTRAL, we introduce weighted ASTRID, incorporating gene tree uncertainty into ASTRID. ASTRID, a fast and more accurate variant of NJst, is based on the internode distance, defined by ASTRID as the number of edges between two taxa in a gene tree. We explore variations of this internode distance where branch uncertainty is considered. Notably, ASTRID is shown to have competitive accuracy against ASTRAL [7, 10] while having a much faster running time [10, 11], both of which we hope to generalize to weighted ASTRID when compared against weighted ASTRAL, obtaining a fast alternative to a very accurate method.

The rest of the study is organized as follows. We begin with a description of weighted ASTRID and introduce the two ways of weighting the internode distance matrix. We then describe our experimental study, choosing parameters for weighted ASTRID and comparing it to other methods, followed by the results for our experimental study using both simulated and biological data. These results show in general that weighted ASTRID improves ASTRID accuracy under most conditions, and our implementation of weighted ASTRID is much faster than the unweighted ASTRID code. We also find that weighted ASTRID is faster than ASTRAL and weighted ASTRAL, and is competitive for accuracy with these methods. We conclude the paper with a discussion of our observations and directions for future work.

## Materials and methods

### Basic definitions

Let *n* denote the number of taxa and let *k* denote the number of genes assuming a set of gene trees. Given an unrooted phylogenetic tree *T*, we denote its leafset by ${\mathcal {L}}(T)$ and its edge-set by *E*(*T*). For each edge *e* in *E*(*T*), deleting *e* from *T* partitions the leaves into two sets defined by the two connected components separated by *e*; we denote this bipartition by $\pi _e$, and we denote the set of bipartitions of *T* by $C(T) = \{\pi _e \,|\, e \in E(T)\}$. We note that *C*(*T*) uniquely defines the (unrooted) topology of *T*. Note that the terms “branch” and “edge” have the same meaning, and we use both in this document.

A bipartition $\pi _e$ is said to be trivial if *e* is incident to a leaf, since in such case $\pi _e \in C(T)$ for any *T* on the same leafset. A tree *T* is said to be a contraction of $T'$ if $C(T) \subset C(T')$. The Robinson-Foulds distance (RF distance) [28] between two trees *T* and $T'$ on the same leafset is the size of the symmetric difference between the bipartitions of *T* and $T'$, i.e., $|C(T) \, \triangle \, C(T')|$. Given two binary trees *T* and $T'$, we define the nRF (error) rate as their RF distance normalized by $2 n - 6$ (the number of non-trivial bipartitions), obtaining a value that is between 0 and 1.

For taxa $u, v \in {\mathcal {L}}(T)$, let $P_T(u, v)$ denote the set of edges on the unique path connecting *u* and *v* in *T*. Given an estimated gene tree *G*, we assume that each internal edge *e* is associated with a branch support value *s*(*e*) denoting the confidence that this edge is correctly estimated, where $s(e) \in [0, 1]$. We say an edge is correctly estimated (in topology) if the bipartition associated with that edge is present in the true gene tree, thus $s(e) = 1$ if *e* is incident to a leaf. We also let *l*(*e*) denote the length of the edge *e*, normally given in substitution units.

### NJst and ASTRID: distance-based summary methods

One approach to species tree estimation is based on combining estimated gene trees, using a distance matrix computed for the input gene trees, and then applying a distance-based method, such as neighbor joining [29]. In this study, we will assume that any discordance between the true gene tree and the true species tree is due to incomplete lineage sorting, and so all gene trees are single copy. We do not assume that the gene trees contain all the species, however, and we also assume that the gene trees are unrooted.

*NJst* In 2011, the NJst method was developed, which uses this approach [30]. In NJst, the distance between two taxa *i* and *j* within a tree is the number of nodes on the path between the leaves for those taxa. This “internode distance” is then averaged across all the gene trees, thus defining the “average internode-distance matrix”. Given the distance matrix, neighbor joining [29] is then used to compute a tree. This method, referred to as NJst, is polynomial time and statistically consistent under the MSC [31].

The proof that NJst is statistically consistent under the MSC for this approach has two parts: first, that the average internode distance matrix converges, as the number of genes increases, to an additive matrix for the species tree, and second, that neighbor joining return the species tree when the average internode distance matrix is close enough to this additive distance matrix. The proof that the average internode distance matrix converges to an additive matrix for the species tree was provided in [31], and the guarantee for neighbor joining returning the species tree when the estimated distance matrix is close enough to an additive matrix was proven in [32]. This last condition is referred to by saying that neighbor joining has a “positive safety radius”, which means that for the true tree *T* with additive matrix *D*, there is some value $\epsilon >0$ so that when given a distance matrix *d* such that $L_{\infty }(d,D) < \epsilon$, then neighbor joining is guaranteed to return the unrooted topology of *T*.

*ASTRID* Note that the proof that NJst is statistically consistent under the MSC enables any distance-based method to replace neighbor joining, as long as the substitute method also has a positive safety radius. Once such method is balanced minimum evolution (BME), which also has a positive safety radius [33, 34]. Moreover, BME has several theoretical properties that suggest that it will have a better sample complexity (and hence better accuracy) than neighbor joining (see discussion about the edge safety radius in [34]). This observation led to the development of ASTRID, which we now describe.

ASTRID is very similar to NJst in design in that it computes a distance matrix from the gene trees and then computes a tree on the distance matrix. The major difference between ASTRID and NJst is that it uses a heuristic for BME available in FastME [35] to construct a species tree from the distance matrix it computes instead of neighbor joining. A minor difference between ASTRID and NJst is that ASTRID uses a slightly different distance matrix: instead of using the number of nodes in a gene tree between the leaves for each pair of taxa, it uses the number of edges. It is trivial to see that this way of defining the distance matrix also converges to an additive matrix for the species tree; hence, this change does not impact statistical consistency. Because the matrix it computes is not exactly the same, we will refer to it as the intertaxon distance matrix.

Like NJst, ASTRID is statistically consistent under the MSC. However, the proof for statistical consistency for ASTRID is somewhat more complicated than for NJst, since ASTRID uses a heuristic search within FastME for BME, which is NP-hard [36]. This heuristic search begins with a greedy BME tree, followed by either NNI (nearest-neighbor interchange) or SPR (subtree prune and regraft) moves, until a local optimum is found. Although this heuristic uses a local search strategy, this approach is guaranteed to converge to the true species tree topology when the input distance matrix is sufficiently close to the additive matrix for the species tree (i.e., like neighbor joining, it also has a positive safety radius) [33, 34]. Thus, we have the following theorem:

#### Theorem 1

ASTRID is statistically consistent under the MSC, provided that the default heuristic is used and run until convergence to a local optimum.

Several simulation studies have shown ASTRID has accuracy that can be comparable to that of ASTRAL and can scale to large datasets [7, 10]. Moreover, early implementations of ASTRID were shown to be much faster than early versions of ASTRAL on large datasets [10], and although ASTRAL has continued to improve in speed, there may still be a runtime advantage to ASTRID. Finally, ASTRID is a key technique used in FASTRAL for speeding up ASTRAL.

Here we describe how we run ASTRID on the datasets in this study. We begin with some notation that we will use throughout this paper.

*Notation* We let *G* denote a gene tree in the set ${\mathcal {G}}$ of gene trees. For *u*, *v* two taxa, we let $d_G(u, v)$ denote the number of edges in the path $P_G(u, v)$ between the leaves for *u*, *v* in *G*, and we let ${\mathcal {G}}_{u,v}$ denote the set of gene trees that have both *u* and *v*.

*ASTRID given complete distance matrices* We now describe how ASTRID operates when the average intertaxon distance matrix has no undefined entries; thus, we assume that for every pair of taxa *u*, *v* there is some gene tree that has both *u* and *v*, and so ${\mathcal {G}}_{u,v}\ne \emptyset$ for all *u*, *v*. We refer to this as saying that the distance matrix is complete. ASTRID proceeds as follows:

1. For each pair of taxa *u*, *v*, we set *D*[*u*, *v*] to be the empirical mean of $d_G(u, v)$ where *G* ranges over ${\mathcal {G}}_{u, v}$. Thus, we set $D[u, v] = \frac{\sum _{G \in {\mathcal {G}}_{u, v}} d_G(u, v)}{|{\mathcal {G}}_{u, v}|}$.
2. We run FastME’s heuristic for balanced minimum evolution [35], under the accurate setting of using extra rounds of NNI and SPR moves (referred to as FastME from here on) on *D*, outputting an unrooted species tree.

*ASTRID given incomplete distance matrices* To handle incomplete distance matrices, i.e., the case where some pair of taxa *u*, *v* do not appear together in any gene tree and so $|{\mathcal {G}}_{u, v}| = 0$, ASTRID instead first marks the corresponding entry of *D*[*u*, *v*] as “missing” in Step 2. FastME requires complete distance matrices, and as such these missing entries must be imputed.

In the original version of ASTRID [10], this issue was addressed by using methods from the PhyD* family [37] of tree estimation methods, which are specifically designed to handle distance matrices that are incomplete (i.e., entries that are undefined). However, in subsequent research (published in Pranjal Vachaspati’s PhD dissertation [38]), a modification to this approach was found to produce better accuracy, which we now describe:

a Run a variant of UPGMA [39] on *D* (that contains missing entries) called UPGMA$^{*}$. Recall that UPGMA is an agglomerative clustering algorithm defined on the “average” distance between two clusters. In UPGMA$^{*}$, two clusters *A* and *B* are candidates for joining only if there exists taxa $u \in A$ and $v \in B$ such that the distance between *u* and *v* is defined (non-missing), and in such case the average distance between the clusters *A* and *B* is the average of all such distances between such pairs *u*, *v*. The resulting UPGMA$^*$ tree is denoted by $U_1$ and we let $A_1$ be the matrix of path lengths (counting the number of edges) between the leaves in $U_1$.
b We define matrix $D_1$, a completion of *D*, as follows. For each *u*, *v* such that *D*[*u*, *v*] is undefined (because ${\mathcal {G}}_{u,v}=\emptyset$), we set $D[u, v] = A_1[u,v]$. We denote this filled-in matrix by $D_1$, and we note that the matrix $D_1$ is complete.
c We run FastME with only NNI moves on $D_1$, obtaining a tree $U_2$. As before, we let $A_2$ denote the matrix of path lengths for $U_2$, and we complete *D* using $A_2$, thus producing complete matrix $D_2$.

As described, the distance matrix $D_2$ is complete, and can be used as input to any tree estimation method that operates using distances. In [38], FastME trees estimated using this distance matrix were more accurate than trees estimated using methods from PhyD* applied to the incomplete distance matrices given as input. This way of running ASTRID is available in the GitHub site [40], where it is referred to as ASTRID-2.

### Weighted ASTRID

In the definition of the intertaxon distance, each edge contributes equally to the intertaxon distance for each pair of taxa it separates. Intuitively, under the realistic assumption that gene trees are estimated with a non-trivial amount of error, some branches will be more reliably estimated than the others. As such it makes sense to assign weights to branches as some confidence of them correctly contributing to the intertaxon distance. The branch lengths could also be used as such a proxy, because short branches are empirically hard to estimate. Thus, our problem becomes to choose appropriate weighting schemes for the edges based on information already annotated in the gene trees, that is, the branch support and branch lengths. Because branch support is already designed as some statistical confidence of the correctness of some branch, it seems natural to naively assign the support directly as the weight for each branch. We alternatively explore simply assigning the branch length as the weight. The details are presented as follows.

#### Distance defined by branch support

We now formally introduce **wASTRID-s** (weighted ASTRID by support), analogous to the naming of weighted ASTRAL by support. Here we try one simple approach, defining each branch’s contribution to the intertaxon distance as its support instead of 1, which gives rise to the following definition of $d_G(u, v)$, the new support-weighted intertaxon distance replacing the intertaxon distance from step 2 of ASTRID:

$$\begin{aligned} d_G(u, v) = \sum _{e \in P_G(u, v)} s(e) \end{aligned}$$

In reality, several different measures of support exist with different running time and accuracy trade-offs [41]. As reported in [25], the approximate Bayesian support [41] of IQ-TREE led to the most accurate species tree reconstruction, although other measures of support also led to accuracy improvements over unweighted ASTRAL. We leave this choice of support as a parameter to be decided later for wASTRID-s in an experiment for parameter exploration (Experiment 1).

#### Distance defined by branch lengths

Unlike branch support, which is designed to be a measure of statistical confidence on the correctness of a branch, branch lengths can only serve as proxies to such information, where shorter branches are empirically harder to estimate likely as a result of shorter branches containing less information (fewer substitutions) [42]. We do not attempt a complex conversion here, and simply just assign the branch length as the confidence similar to how we use the support values:

$$\begin{aligned} d_G(u, v) = \sum _{e \in P_G(u, v)} l(e) \end{aligned}$$

Notably, this definition of $d_G(u, v)$ coincides with STEAC [43] (motivated by a different perspective of the coalescence time between genes), and potentially under a more accurate setting when paired with the FastME step of ASTRID. In addition, we also explore whether and how to normalize the input branch lengths of the gene trees for the weighting. We name this final algorithm **wASTRID-pl** (weighted ASTRID by path-lengths).

### Running time

The runtime for wASTRID-s and wASTRID-pl has two parts: the calculation of the distance matrix and then running FastME. Here we derive the runtime for each part.

#### Theorem 2

The average intertaxon distance matrix of wASTRID-s and wASTRID-pl can be obtained in $O(k n^2)$ time, where *n* is the number of species and *k* is the number of genes. The runtime using FastME on this input matrix, and using *p* NNI or SPR moves, is $O(pn^2)$. Hence, the total runtime is $O((k + p) n^2 )$.

#### Proof

We begin by demonstrating that the asymptotic runtime for calculating the distance matrix is the same for wASTRID-s, wASTRID-pl, and ASTRID. For each gene tree, our metricization simply assigns already-computed values as lengths to each edge; thus, using dynamic programming, calculating the intertaxon distance across all pairs of taxa per gene tree takes $O(n^2)$ time (for normalizing branch lengths in wASTRID-pl, we only explore ways to normalize that do not affect this asymptotic running time). Hence, the average intertaxon distance matrix can be calculated in $O(k n^2)$ time.

The runtime for FastME, given the distance matrix, depends on the number *p* of NNI or SPR moves. According to [35, 44], FastME optimizing BME, using NNI or SPR moves, uses $O(n^2)$ for the starting tree and $O(n^2 + pn \times diam(T))$, where *diam*(*T*) is the topological diameter of the output tree and *p* is the number of moves, for the heuristic search. Since $diam(T) \le n-1$, this is $O(pn^2)$. $\square$

We also re-implemented the calculation of the distance matrix to improve empirical runtime compared to the available version of ASTRID in [40]. The asymptotically optimal algorithm is easy to devise because the naive algorithm, which, given a gene tree, starts a tree traversal at each leaf to obtain the all-pairs intertaxon distance, is already quadratic time per gene and also asymptotically optimal due to each gene tree having $$\left( {\begin{array}{c}n\\ 2\end{array}}\right)$$ distances. The original ASTRID implementation, in this vein, uses an algorithm which implicitly performs multiple traversals in the tree. We instead implemented an intertaxon-distance algorithm from TreeSwift [45] based on post-order traversal, through which we hope to achieve better empirical performance due to better cache locality in its simultaneous maintenance of multiple distances from the leaves in an array. This produces for us a speed advantage for wASTRID as well as an alternative way of running ASTRID, which we refer to as ASTRID-3.

## Experimental study

### Overview

We conduct five experiments. The first experiment is parameter exploration and uses training data; all subsequent experiments use the testing data, which are disjoint from the training data.

- Experiment 1: we explore parameter choices (choice of branch support for wASTRID-s and normalization scheme for wASTRID-pl) for weighted ASTRID.
- Experiment 2: we compare the accuracy and running time of weighted ASTRID against other methods on a diverse set of simulated conditions, where all genes are complete, and so have all the species.
- Experiment 3: we compare ASTRID, wASTRAL-h, and the best variant of wASTRID (as determined by previous experiments) on the Jarvis et al. [46] avian biological dataset.
- Experiment 4: we explore weighted and unweighted ASTRAL and ASTRID on datasets where the average intertaxon distance matrix might be incomplete (i.e., where there is at least one pair of taxa that do not appear together in any gene tree). Specifically, we explored two models of missing data, one uniformly deleting a fixed number of taxa across gene trees, and also a “clade-based” model of missing data, denoted by $M_{\text {clade}}$ from [47]. Both types of missing data results in gene trees that each almost always has taxa missing.
- Experiment 5: we conducted a detailed running time comparison between ASTRID-2 and ASTRID-3, where ASTRID-3 differs from ASTRID-2 by a faster implementation for calculating the average intertaxon distance matrix.

### Datasets

**Table 1:** Dataset statistics. All but the avian dataset from [46] are simulated datasets, with known true gene trees and species trees. The ILS levels of the datasets are categorized according to their AD percentages, where below $25\%$ is low ILS (L), between $26\%$ and $39\%$ mid ILS (M), between $40\%$ and $59\%$ high ILS (H), and higher AD very high ILS (VH). SH-like denotes FastTree default support; BS denotes standard bootstrap support using FastTree or RAxML; aBayes denotes IQ-TREE approximate Bayesian support

Dataset	# taxa	# genes	# reps	AD % (ILS)	Branch support	Exp.
ASTRAL-II MC2 [13]	201	1000	10	33 (M)	SH, BS, aBayes	1
ASTRAL-III S100 [14]	101	1000	50	46 (H)	aBayes	2,5
$M_{\text {clade}}$ missing data	101	200	50	46 (H)	aBayes	4
*iid* missing data	101	200	50	46 (H)	aBayes	4
ASTRAL-II MC3 [13]	201	1000	50	21 (L)	aBayes	2
ASTRAL-II MC5 [13]	201	1000	50	34 (M)	aBayes	2,5
ASTRAL-II MC1 [13]	201	1000	50	69 (VH)	aBayes	2
ASTRAL-II MC6H [48]	201	1000	50	9 (L)	aBayes	2
ASTRAL-II MC11H [48]	1001	1000	50	35 (M)	aBayes	2,5
Avian 2x [23]	48	1000	20	29 (M)	aBayes	2
Avian 1x [23]	48	1000	20	47 (H)	aBayes	2
Avian 0.5x [23]	48	1000	20	60 (VH)	aBayes	2
Mammalian 2x [23]	37	200	20	21 (L)	aBayes	2
Mammalian 1x [23]	37	200	20	29 (M)	aBayes	2
Mammalian 0.5x [23]	37	200	20	50 (H)	aBayes	2
Jarvis et al. avian [46]	48	14446	1	not known	BS	3

We assembled a set of diverse data from prior studies (see Table 1), consisting of various simulated conditions with estimated gene trees and one biological dataset (“avian biological”) from the avian phylogenomics project [46]. We separate the datasets into training (Experiment 1) and testing (Experiments 2–5).

We use the nomenclature of the original ASTRID study and refer to the SimPhy-simulated datasets from the ASTRAL-II study [13] by an “MC” name (where “MC” refers to “model condition”). We additionally replaced some ASTRAL-II datasets by some “H” variants generated by a prior separate study [48] to induce a level of GTEE closer to the rest of the conditions, which span from 23% to 55%. The ILS levels of the datasets are measured in average discordance (AD), defined as the average nRF rate between the true species tree and the true gene trees. Like the original ASTRID study [10], we classify the ILS levels of the datasets into four categories according to their AD values, where below $25\%$ is classified as low ILS (L), between $26\%$ and $39\%$ medium ILS (M), between $40\%$ and $59\%$ high ILS (H), and higher AD considered very high ILS (VH). For the simulated conditions, we subsample *k* of the gene trees with $k = 50, 200, 1000$, except for the mammalian simulation where we sample $k = 50, 100, 200$ instead, as only 200 gene trees were provided.

In Experiment 4, we evaluate methods on datasets where some gene trees are missing one or more species. For this experiment, we created missing data variants of the S100 (seqlen = 400) dataset under two models of missing data: the *i.i.d.* model and the $M_{\text {clade}}$ model. For the *i.i.d.* case, we deleted a fixed number of taxa ($20\%, 40\%, 60\%, 80\%$ of the total number of taxa) from each gene; thus, every gene tree is incomplete (i.e., misses at least one species). For the $M_{\text {clade}}$ model, the process is more complicated. Briefly, each gene tree uniformly samples a clade from the species tree above a certain size (a parameter *x* relative to the number of taxa in the species tree), and only retains taxa from this clade. We varied *x* in 0.2, 0.4 and 0.6, which resulted in an average percentage of taxa being deleted of $46\%, 23\%$ and $10\%$, respectively for the $M_{\text {clade}}$ model. Thus, for the $M_{\text {clade}}$ model, it is *possible* for a gene tree to be complete (i.e., have all the species), but not very likely for this to occur.

We now describe how we compute branch support in our experiments. The weighted ASTRAL study [25] provided gene trees reannotated with aBayes support [41] and edge lengths inferred using IQ-TREE [49] for the ASTRAL-II and ASTRAL-III datasets. We also reannotated the avian and mammalian simulation with aBayes support and IQ-TREE edge lengths, because aBayes was determined as the best measure of support also for wASTRID-s. We use the MC2 condition as the training data for both wASTRID-s and wASTRID-pl, where to explore the choice of edge support for wASTRID-s, we took the original gene trees estimated using FastTree [50] and estimated branch support using two techniques: the default FastTree SH-like support and also bootstrap support [51] using 100 bootstrap trees. For Experiment 4, we only used the IQ-TREE aBayes support, computed on the reduced gene trees and using the reduced alignments. See Appendix for additional discussion about branch support.

### Methods

We ran weighted and unweighted versions of ASTRAL and ASTRID.

- ASTRID (v2.2.1), available at [40]. With the exception of Experiment 4, we turn off missing data imputation.
- wASTRID, available at [52], missing data imputation will be automatically turned on by the software when necessary.
- ASTRAL(-III) (v5.7.8), available at [53]. Although ASTRAL-III enables analyses of gene trees with low-support branches collapsed, we use the fully resolved gene trees as input, as this allows us the fully explore the impact of weighting.
- wASTRAL-h (hybrid weighted ASTRAL, v.1.4.2.3). This was the most accurate version of weighted ASTRAL from the original study [25], using both branch lengths and support to weight gene tree quartets. wASTRAL-h supports parallelization, so we run wASTRAL-h with 16 threads. wASTRAL-h is available as part of the ASTER [54] software suite.

For all analyses using wASTRID and ASTRID, when we ran FastME to search for a BME solution, we ran FastME with the most accurate setting that uses the same greedy BME starting tree and follows with both NNI and SPR moves to improve the score.

Many of the datasets in our study have FastTree-inferred gene trees that were reannotated with IQ-TREE approximate Bayesian support. FastTree-inferred trees have polytomies when the input has identical sequences, but these polytomies will be resolved when the trees are reannotated by IQ-TREE. Since polytomy resolution may add false positive edges that adversely affect the accuracy of the unweighted methods, in these cases, we run the unweighted methods on the original FastTree gene trees.

### Evaluation criterion

For simulated datasets, we report the topological error rate of the reconstructed species trees using the normalized Robinson-Foulds error (nRF error) [28] with respect to the true species trees, where the nRF error rate is the number of bipartitions in the estimated and true species trees that do not appear in both trees, normalized by the total number of non-trivial bipartitions in the two trees. Because all the inferred and true species trees are binary, the nRF error rate is the same as the missing branch rate (i.e., the fraction of the non-trivial bipartitions in the true species tree that are missing from the reconstructed tree). For these calculations, the non-trivial bipartitions of a tree are defined by the internal edges in the tree, i.e., the edges that are not incident to leaves.

On the avian biological dataset, as the true tree is not known, we compare the estimated species trees against prior topologies (wASTRAL tree and published trees). We also compute the local posterior-probability (localPP) branch support [55] for the reconstructed species trees obtained using wASTARL-h to assess the reliability of the branches.

On all datasets, we keep track of the wall-clock running time of the methods, the time taken from consuming the input gene trees (that may have been preprocessed with new branch support values) until outputting the species tree.

### Experimental environment

All experiments before Experiment 5 were conducted on the Illinois Campus Cluster, a heterogeneous cluster that has a four-hour running time limit. The heterogeneity of the hardware makes the wall-clock running times not directly comparable across runs, but can still be used to gather obvious running time trends.

All runs in Experiment 5 were performed serially on an Apple M1 Macbook Pro (model Z11B000E3LL/A) to ensure accurate comparison of running times.

## Results

### Experiment 1: parameter selection

**Fig. 1:**
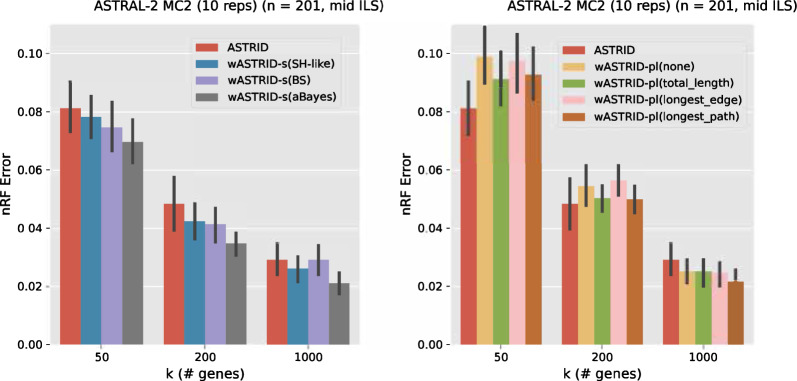
Comparison of the choice of branch support and the choice of branch length normalization strategy for wASTRID-s and wASTRID-pl respectively on the training data, showing the species tree topological error rates (nRF error). “SH-like” is FastTree SH-like support. “aBayes” is IQ-TREE approximate Bayesian support. “BS” is bootstrap support using 100 FastTree trees. The *x*-axis varies in the number of genes *k* in the input. Results are shown averaged across ten replicates. Error bars show standard error

In Fig. 1, we explore the choice of branch support among the default FastTree SH-like support, IQ-TREE approximate Bayesian (aBayes) support (normalized to the [0, 1] range), and bootstrap support (100 FastTree replicates) on the training datasets. The best accuracy of wASTRID-s is obtained by using the normalized aBayes support on gene trees. All measures of support, however, improved the species tree estimation error in general. The superiority of (normalized) aBayes support is consistent with the support chosen for weighted ASTRAL, where it was also found superior to SH-like support and bootstrap support. This advantage is even more pronounced when considering that aBayes support can be obtained much faster than bootstrap support [41].

On this training dataset, wASTRID-pl attained the highest accuracy when normalizing the branch lengths in each gene tree by the maximum path length in that gene tree (better than no normalization). Interestingly, while worse than ASTRID with fewer genes $k \in \{50, 200\}$, wASTRID-pl attained higher accuracy than ASTRID when $k = 1000$. However, when comparing wASTRID-s and wASTRID-pl, wASTRID-pl was always less accurate.

### Experiment 2: results on simulated datasets

In this experiment, we show four-way comparisons among ASTRID, ASTRAL, weighted ASTRAL (wASTRAL-h), and weighted ASTRID (wASTRID-s). We additionally show wASTRID-pl on S100 but omit showing its results later as wASTRID-pl was discovered to be on all datasets less accurate than wASTRID-s. We put an emphasis on the accuracy (nRF error), while later revisiting the problem of running time.

#### ASTRAL-III S100

**Fig. 2:**
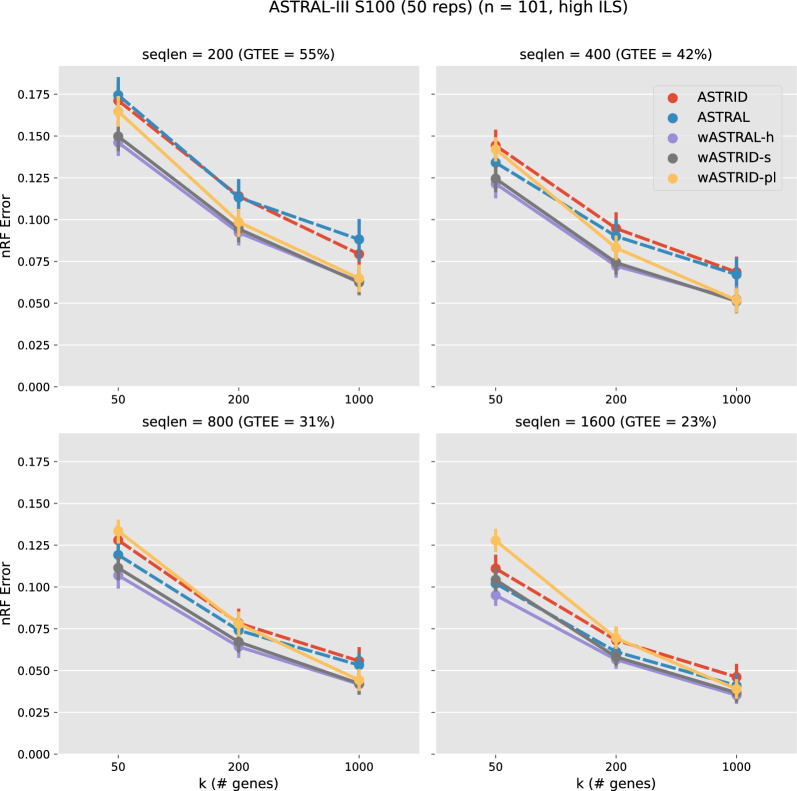
Topological error of species tree across methods on the ASTRAL-III S100 dataset ($n = 101, \text {AD} = 46\%$). Subfigures vary the sequence lengths, affecting the gene tree estimation error (measured in GTEE, the average distance between estimated gene trees and true gene trees). The *x*-axis varies in the number of genes. Results are shown averaged across 50 replicates with standard error bars. All weighted methods (wASTRID, wASTRAL) ran on gene trees reannotated with IQ-TREE aBayes support branch support and lengths. All methods achieve better accuracy when given more gene trees (larger *k*) or more accurate gene trees (lower GTEE). Weighted methods are more accurate than unweighted ones. wASTRID-s and wASTRAL-h have almost the same accuracy

This 101-species dataset contains four conditions that varied in the gene tree estimation error (GTEE, measured by the average nRF error between the estimated gene trees and their corresponding true gene trees) by varying the sequence lengths. We show the results in Fig. 2. We show the unweighted methods (i.e., ASTRID and ASTRAL) in dotted lines.

Many trends are as expected. For example, summary methods become more accurate as *k* (the number of genes) increases, and all methods also improve in accuracy when given more accurate estimated gene trees. These two trends are unsurprising and well-documented across studies for summary methods in general (e.g., [7]).

More interestingly, the weighted methods (wASTRID-s, wASTRAL-h) are clearly more accurate than their unweighted counterparts, especially at higher levels of GTEE (GTEE $= 0.55, 0.42$). The improvement in accuracy from the weighted methods does not seem to depend on the number of genes, suggesting that the noise brought by low-quality gene trees is not resolved by having ample data. This advantage of the weighted methods, however, is smaller as more accurate gene trees are used (GTEE = 0.31), as expected.

wASTRID-s clearly improves upon ASTRID on this dataset, across all conditions and all numbers of genes. wASTRID-s notably almost matches the accuracy of wASTRAL-h. With $k \in \{200, 1000\}$, no clear benefit exists for using wASTRAL-h on the shown conditions.

On this dataset, wASTRID-pl, similar to trends seen in the training dataset, attains better accuracy than ASTRID when $k = 1000$, but is almost always worse than wASTRID-s.

#### ASTRAL-II SimPhy

**Fig. 3:**
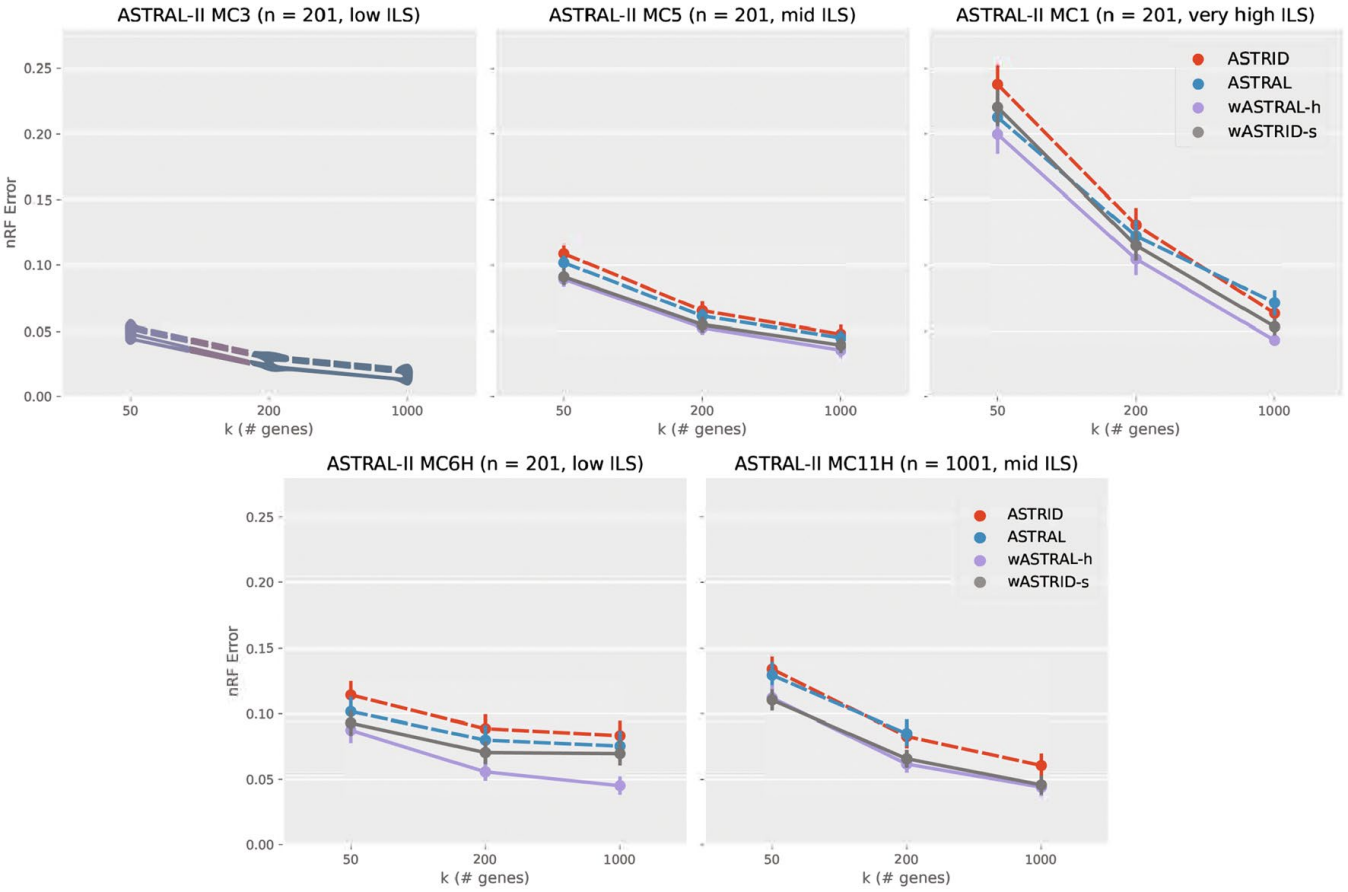
Topological error (nRF error rate) of species tree across methods on selected conditions on the ASTRAL-II SimPhy conditions ($n = 201, 1001, 201$, $\text {AD} = 21, 35, 69\%$ respectively). Each subfigure depicts a different model condition. The *x*-axis varies in the number of genes. Results are shown averaged across 50 replicates with standard error bars. ASTRAL did not finish 24 out of the 50 replicates within four hours for $k = 1000$ on MC11H and thus the data point was omitted. All weighted methods (wASTRID, wASTRAL) were run on gene trees reannotated with IQ-TREE aBayes support branch support and lengths. Weighted methods are more accurate than unweighted ones. wASTRID-s on MC1 was less accurate than wASTRAL-h and otherwise has the same accuracy

We show the results of the ASTRAL-II SimPhy datasets in Fig. 3. Across all conditions in this dataset, the weighted methods are more accurate than the unweighted methods. This advantage does not seem to depend on the level of ILS or the number of species. Even under the easiest condition (MC3), wASTRAL-h and wASTRID-s still consistently achieved better accuracy. All methods also performed worse in accuracy as ILS increased, as expected.

While wASTRID-s still consistently improved upon ASTRID in accuracy on this dataset, we also see datasets where wASTRID-s is worse than wASTRAL-h. The relative performance of wASTRID-s and wASTRAL-h seems related to the relative performance of the base methods: MC1 and MC6H are the two conditions that ASTRAL is in general more accurate than ASTRID, but the relative performance of the base methods does not explain the whole picture – for MC1 going to $k = 1000$, ASTRID became more accurate than ASTRAL yet wASTRID-s is still worse than wASTRAL-h. More positively, on the other conditions of this dataset, wASTRAL-h has nearly the same accuracy as wASTRID-s, although wASTRAL-h is marginally more accurate, which might be due to the hybrid weighting of wASTRAL-h, which also incorporates the branch lengths for better accuracy.

The model condition with the largest number of species, i.e., MC11H with 1001 species, presented computational challenges for ASTRAL. Specifically, on roughly half of the replicates of model condition MC11H with $k = 1000$ genes, ASTRAL did not finish under our four-hour time limit (see Appendix Sect. "Failures to complete" for more details), but wASTRAL-h did.

#### Avian and mammalian simulations

**Fig. 4:**
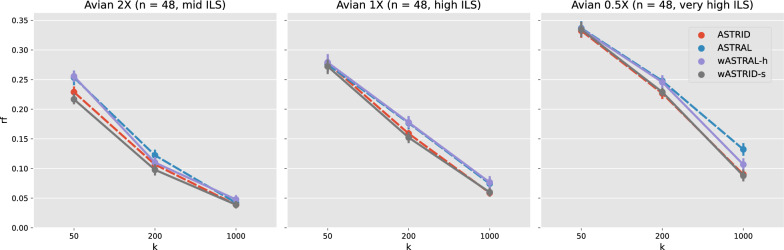
Topological error (nRF error rate) of species tree across methods on the avian simulation ($n = 48$). Each subfigure depicts a different model condition. The *x*-axis varies in the number of genes. Results are shown averaged across 20 replicates with standard error bars. All weighted methods (wASTRID, wASTRAL) ran on gene trees reannotated with IQ-TREE aBayes support branch support and lengths. ASTRID and wASTRID-s are more accurate than ASTRAL and wASTRAL-h, with a slight accuracy advantage to the weighted methods over the unweighted ones

**Fig. 5:**
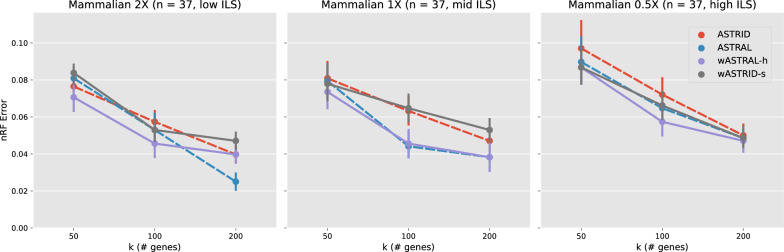
Topological error (nRF error rate) of species tree across methods on the mammalian simulation ($n = 37$). Each subfigure depicts a different model condition. The *x*-axis varies in the number of genes. Results are shown averaged across 20 replicates with standard error bars. All weighted methods (wASTRID, wASTRAL) ran on gene trees annotated with IQ-TREE aBayes support branch support and lengths. ASTRAL and wASTRAL-h are more accurate than ASTRID and wASTRID-s. The weighted methods have mixed accuracy compared to the unweighted ones

The avian and mammalian simulation have model trees inferred on biological datasets. Both datasets have three conditions with varying ILS by scaling the model tree branch lengths by 2X, 1X, or 0.5X, with shorter branch lengths leading to higher degrees of ILS. Notably, prior results [10] showed that ASTRID outperformed ASTRAL on the avian simulation in accuracy, while on the mammalian simulation ASTRAL was more accurate. Also, the mammalian simulation only has 200 genes available, so we vary *k* among 50, 100, 200 unlike the other datasets.

On the avian simulation (Fig. 4), aside from obvious trends (ILS increases difficulty; more genes leads to more accurate reconstruction), same as the original study [10], ASTRID is consistently more accurate than ASTRAL. Strangely, although the weighted methods inherit the relative performance of their base methods, in a few cases the weighted methods do not help in accuracy, but they do not erode accuracy either. Even on conditions where the weighted methods improved accuracy, the improvement was small. For example, wASTRAL-h, even though improving upon ASTRAL, is even less accurate than ASTRID, whereas on previously shown data wASTRAL-h was consistently the best in accuracy. This avian simulation does carry substantial GTEE ($> 50\%$), so it is not clear what led to the weighted methods underperforming.

The results for the mammalian simulation (Fig. 5) paint a more perplexing picture. On the 2X condition, surprisingly, the weighted methods are less accurate than their unweighted counterparts in general. This trend continues with the 1X condition, where wASTRAL-h only mostly matches ASTRAL in accuracy, and wASTRID-s is worse than ASTRID in accuracy. Only on the 0.5X condition do both weighted methods clearly help in accuracy. wASTRAL-h is clearly better than wASTRID-s on this dataset, but this difference can be explained by the accuracy advantage of ASTRAL on ASTRID. While it is again unclear why the weighted methods underperformed, this dataset is relatively easy, compared to the previously shown datasets, with all methods achieving at most 0.05 nRF error rate at $k = 200$; thus, despite the puzzling relative performance of weighted and unweighted methods, the difference in accuracy among methods is very small.

#### Running time

**Fig. 6:**
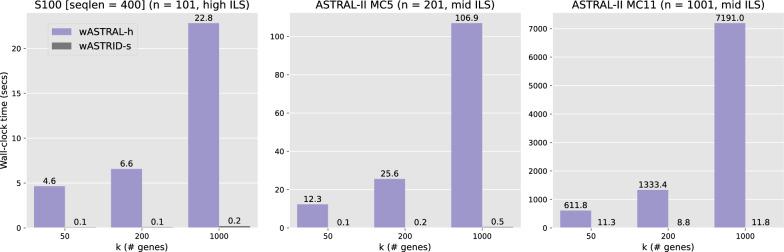
Wall-clock running time (s) comparison of wASTRID-s and wASTRAL-h on selected representative simulated conditions on $n = 101, 201, 1001$ for *k* ranging in 50, 200, 1000. Bars and labels show averages across 50 replicates. wASTRID-s is dramatically faster than wASTRAL-h

**Table 2:** Wall-clock running time (sec) across methods on selected representative simulated conditions on $n = 101, 201, 1001$ for *k* ranging in 50, 200, 1000. Data points show averages across 50 replicates. ASTRAL did not finish on 24 out of the 50 replicates within four hours for $k = 1000$ on MC11H, and thus the data point was omitted. The methods, sorted by the fastest to the slowest, are almost always wASTRID-s, ASTRID, wASTRAL-h, and ASTRAL across all shown conditions. ASTRID and wASTRID-s are much faster than ASTRAL and wASTRAL-h

Running time (s)	k (# genes)	ASTRAL	ASTRID	wASTRAL-h	wASTRID-s
S100 ($n=101$)	50	12.1	0.1	4.6	0.1
	200	29.6	0.2	6.6	0.1
	1000	300.7	1.4	22.8	0.2
MC5 ($n=201$)	50	20.3	0.2	12.3	0.1
	200	57.5	0.6	25.6	0.2
	1000	600.7	1.8	106.9	0.5
MC11H ($n =1001$)	50	552.9	18.0	611.8	11.3
	200	2059.6	14.9	1333.4	8.8
	1000	–	27.5	7191.0	11.8

We show the wall-clock running time of the four methods under three representative conditions ($n = 101, 201, 1001$) in Table 2, with a direct comparison of the two most accurate methods visualized in Fig. 6. While the heterogeneity of the hardware dilutes the comparability of the running times, clearly wASTRID-s and ASTRID are much faster than wASTRAL-h and ASTRAL, with wASTRID-s on average taking less than 12 s even on the largest input, whereas on the same input wASTRAL-h on average takes roughly two hours. In general, wASTRID-s is around two orders of magnitude faster than wASTRAL-h. Although we note that the default flags of both ASTRAL and wASTRAL-h (that we used in the experiments) also calculate support and lengths for reconstructed species tree, in practice, this is a fast step relative to the species tree reconstruction, and does not affect our running time analysis in any substantial way. On MC11H, wASTRID-s and ASTRID took less time going from $k = 50$ to 200, likely due to the $k = 50$ species trees having larger diameters, negatively impacting the FastME step running time which has a linear dependency on the diameter of the output tree.

The weighted methods are faster than their unweighted counterparts. For example on S100 (seqlen $= 400$) with 1000 genes, wASTRAL-h is more than ten times faster than ASTRAL. In addition ASTRAL did not finish for approximately half of the datasets for the largest input (MC11H, $k = 1000$). Aside from the benefit of parallelization (we ran wASTRAL-h using 16 threads, but off-the-shelf ASTRAL does not support parallelization), this speed advantage under a large number of genes of wASTRAL-h over ASTRAL can also be attributed to the algorithmic change implemented in wASTRAL-h. The new weighted ASTRAL algorithm removes the in-practice quadratic dependency of ASTRAL’s search algorithm on the number of genes [56] and instead has a linear dependency on *k* for the running time. wASTRID-s is consistently faster (at least two times faster on most conditions) than ASTRID, showing that the new distance calculation algorithm implemented is more efficient than the original one.

**Fig. 7:**
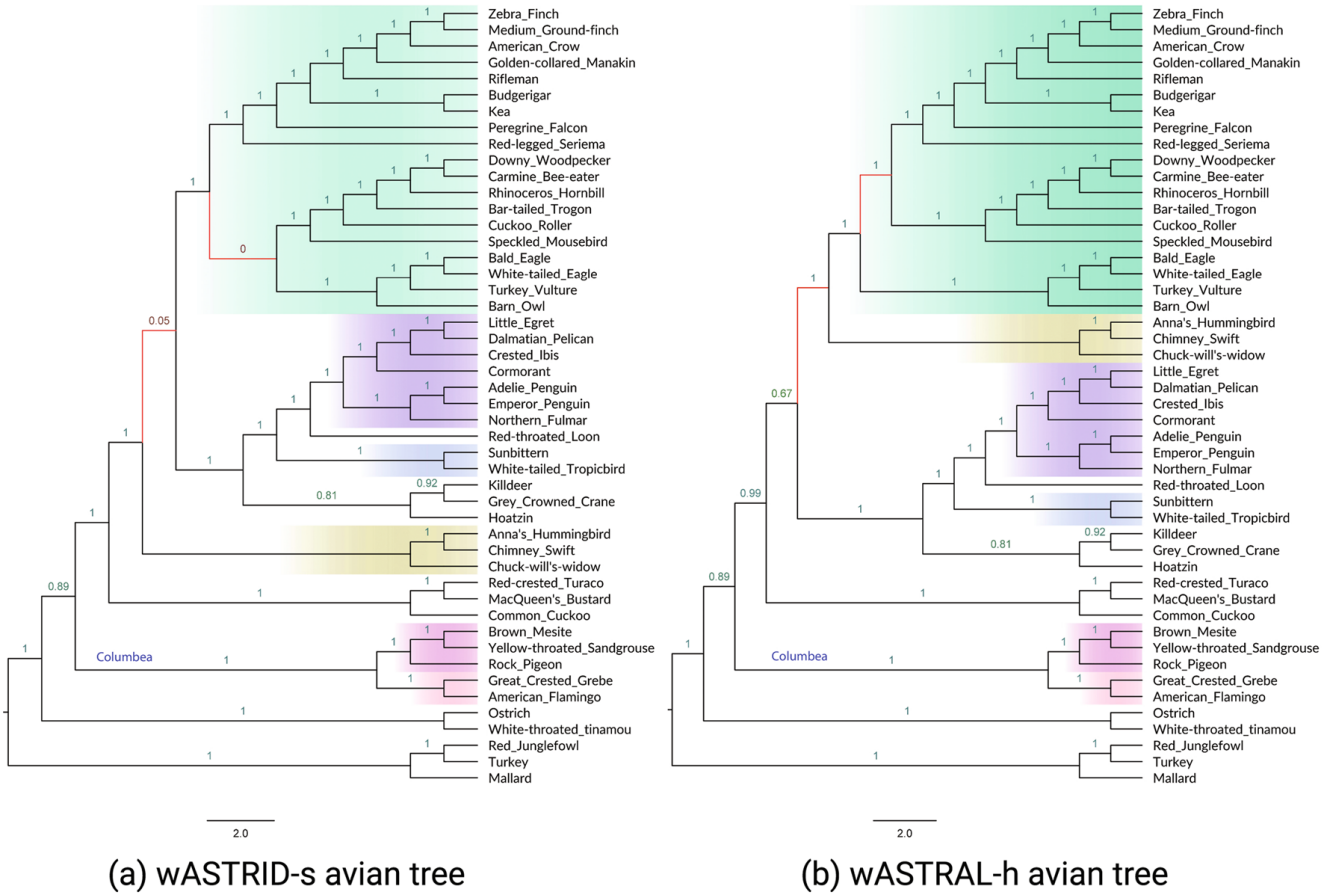
Results on the avian biological dataset ($n = 48, k = 14446$). In (**a**) and (**b**), we show the reconstructed species tree topology of wASTRID-s and wASTRAL-h, annotated with local posterior-probability branch support (localPP) computed using wASTRAL-h. The red branches show where the two trees differ. The six well corroborated clades according to Braun and Kimball [57] are displayed by both trees and highlighted in gradients. For wASTRID-s, the red branches also coincide with the only two low support branches. Contracting the very low support branches for wASTRID-s arrives at a topology compatible with wASTRAL-h. wASTRAL-h took around 294 s to infer its tree. wASTRID-s was very fast and took 1 s to infer its topology

### Experiment 3: results on the avian biological dataset

Jarvis et al. studied the phylogeny of birds using a dataset on 48 taxa using 14,446 genes [46]. The original gene trees were annotated with RAxML [58] bootstrap support, which we directly use in our wASTRID-s analysis. This dataset is known to have very low gene tree resolution, with the average branch support only $32\%$ [23].

ASTRAL on the original set of gene trees reconstructed a species tree that failed to reproduce a few well-supported clades from prior studies, such as the Columbea clade. Nonetheless, contracting low support branches in the gene trees enabled ASTRAL to construct a very plausible topology in agreement with prior studies [14]. In a subsequent study, Zhang and Mirarab reanalyzed the original gene trees using wASTRAL-h and reconstructed the same topology as ASTRAL running on contracted gene trees [25].

We computed trees using both ASTRID and wASTRID on the Jarvis et al. [46] dataset, and compare them to these other estimated trees. We also compute the number of differing branches between the inferred trees and the two published trees of the Jarvis et al. study [46]: the ExaML-based concatenation tree, called the TENT (“total evidence nucleotide tree”), and the coalescent-based published tree based on the result of running MP-EST with statistical binning [23] (i.e., the MP-EST* tree). We also examine whether these trees include the six clades that are proposed by Braun & Kimball to be strongly corroborated [57] for the avian phylogeny.

**Fig. 8:**
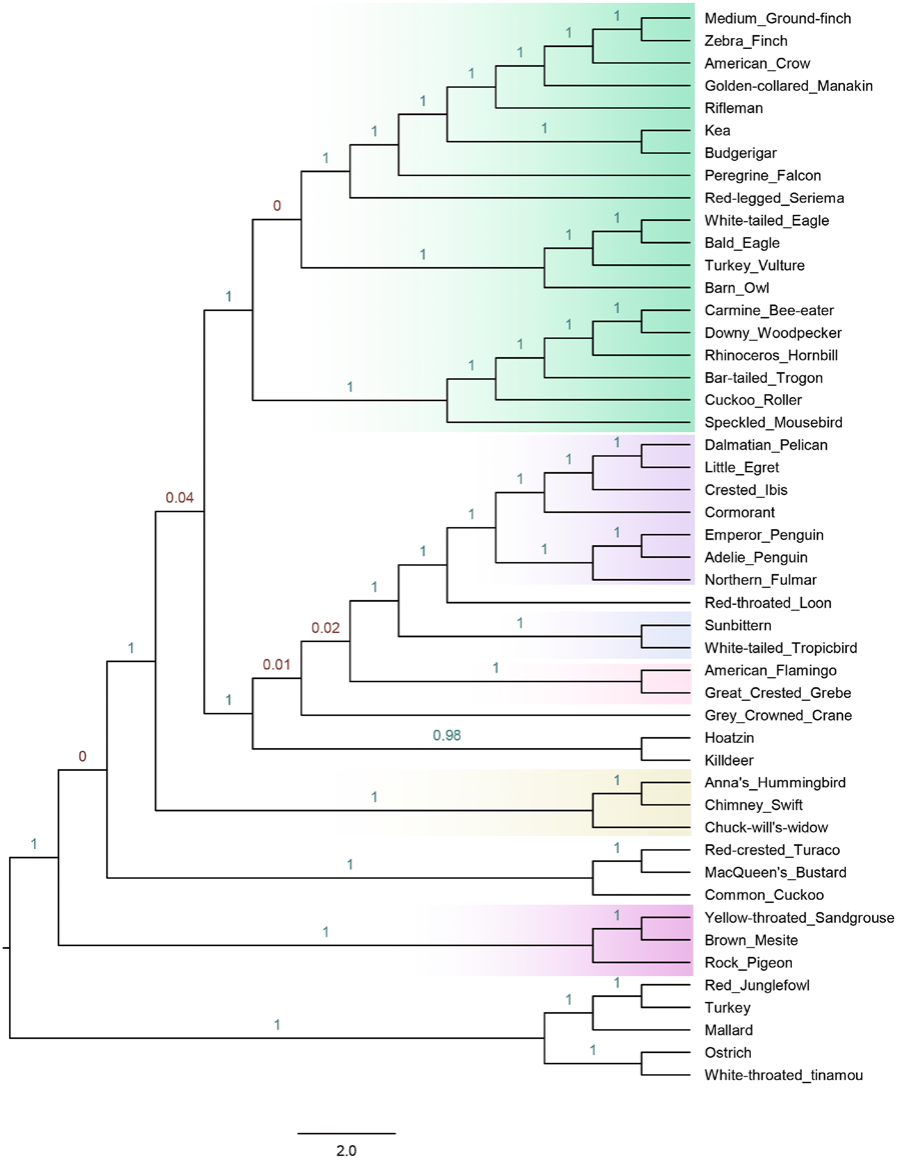
Reconstructed species tree on the Jarvis et al. avian biological data using ASTRID. Branch support values are in localPP values calculated by wASTRAL-h

The wASTRID-s and wASTRAL trees are shown in Fig. 7 and the ASTRID tree is shown in Fig. 8, with branch support given by the localPP criterion. Note that all trees display the six reliable avian clades of Braun & Kimball. Nevertheless, some interesting differences do appear.

The ASTRID tree (shown in Fig. 8) differed in eight or nine edges with each of the wASTRAL-h, TENT, and MP-EST* trees. The ASTRID tree did not recover the Columbea clade, which has also seen strong support in various analyses of this data [14, 46, 59].

Using wASTRID-s, we recovered a topology that is much more in agreement with the other trees, differing in two branches ($4.4\%$ of the branches) with the wASTRAL-h tree. It displays the six well-corroborated clades, and is also in much higher agreement with the published trees (differing in three branches with the TENT, two branches with the MP-EST* tree). Looking closer, the two branches where it differs from the wASTRAL-h tree coincide with the only two extremely low support branches (no more than $5\%$ in bootstrap support), and thus contracting these branches in the wASTRID-s tree arrives at a topology that is a contraction of the wASTRAL-h tree. Both wASTRID-s and wASTRAL-h recover the Columbea-Passerea split, a major conclusion in the original analysis of this data, and even agree on the placement of the hard-to-place Hoatzin.

For running time, both ASTRID and wASTRID-s finished quickly. ASTRID completed in 6.4 s, and wASTRID-s completed in 1.1 s. Our rerun of wASTRAL-h on this data finished in 294.2 s, showcasing the much better scalability of the new weighted ASTRAL optimization algorithm in the number of genes, whereas ASTRAL on the same input took 32 h in [14].

In summary, on this dataset, wASTRID-s inferred a more accurate tree compared to ASTRID, is much faster than wASTRAL-h, and is compatible with wASTRAL-h after contraction of two very low support branches.

### Experiment 4: accuracy given incomplete gene trees

**Fig. 9:**
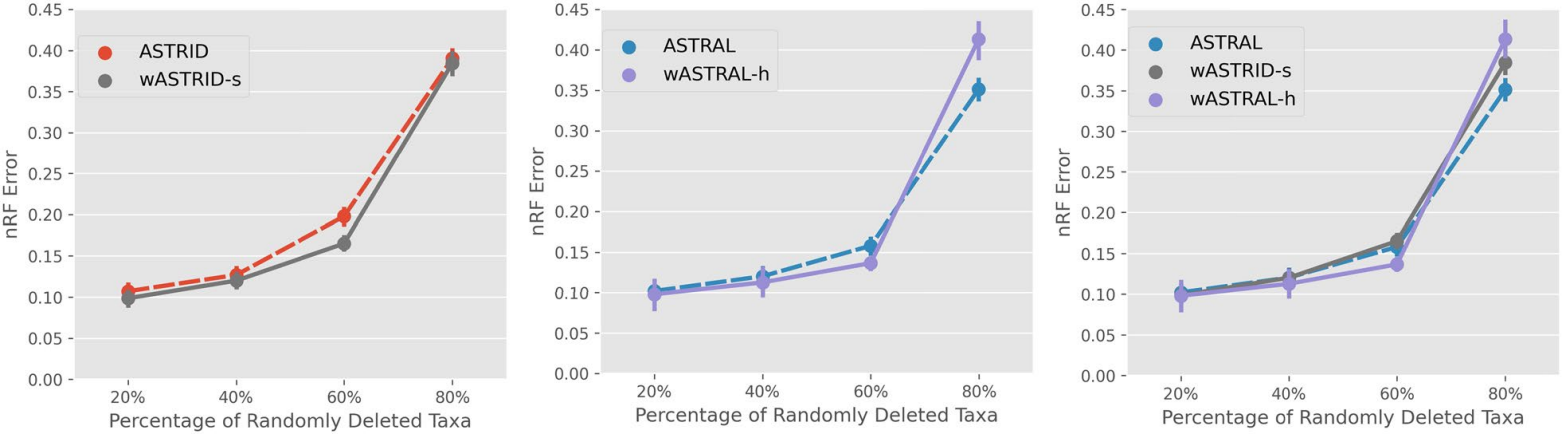
Topological error of species trees estimated for sets of incomplete gene trees due to deletion of a fixed number of taxa on the ASTRAL-III S100 dataset ($n = 101$, AD = $46\%$) given $k=200$ genes with sequences of length 400 (GTEE = $42\%$). Results are shown averaged across 50 replicates with standard error bars. *x*-axis varies in the percentage of taxa deleted across gene trees, under the model where for each gene, a random subset of taxa of a fixed size is deleted from each gene tree

**Fig. 10:**
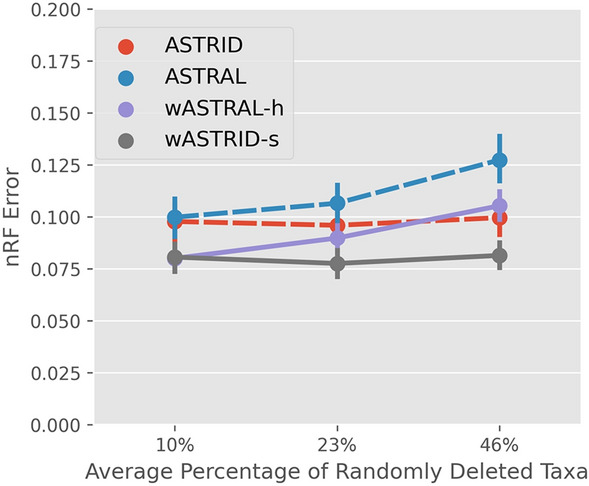
Topological error of species trees estimated for sets of incomplete gene trees due to clade-based missing data, on the ASTRAL-III S100 dataset ($n = 101$, AD = $46\%$) given $k=200$ genes with sequences of length 400 (GTEE = $42\%$), varying the percentage of randomly deleted taxa. Results are averaged across 50 replicates with standard error bars. *x*-axis varies in the average percentage of deleted taxa in the gene trees

In real-world datasets, genes that are incomplete, so that they miss some species, are commonplace. This “missing data” condition can reduce accuracy, as shown in [7, 47], and the impact of this is studied in this experiment.

*Random missing data* We show the impact of missing data using a model of uniformly deleting a fixed number of taxa across gene trees in Fig. 9. As the percentage of missing data increases, all methods increase in error, an expected trend as the amount of information in the input decreases. Comparing the weighted and unweighted versions, weighted methods were more accurate than their unweighted counterparts under low to moderate (20% to 60% taxa randomly missing) missing data. However, under the highest level of missing data (80% taxa missing), ASTRAL achieved the best accuracy across all methods, showing a robustness against extreme missing data while wASTRAL-h achieved the worst accuracy at this extreme. Comparing horizontally between the ASTRID and ASTRAL variants, we see a slight advantage of ASTRAL in accuracy across levels of randomly deleted taxa, suggesting ASTRAL might be more robust to this *i.i.d.* model of missing data. Although we only show results on one condition, our results suggest that the weighted methods still offer accuracy advantages except on the more extreme conditions of missing data, and both ASTRID variants and ASTRAL variants demonstrate reasonable robustness against missing data.

*Clade-based missing data* We show the results under the $M_{\text {clade}}$ based missing data in Fig. 10. Recall that each gene tree uniformly samples a clade from the species tree above a certain size (a parameter $x = 0.2, 0.4, 0.6$ relative to the number of taxa in the species tree), and only retains taxa from this clade. This process resulted in average percentage of taxa being deleted of $46\%, 23\%$ and $10\%$ across the genes.

Under these clade-based missing data conditions, the weighted methods are consistently more accurate than the unweighted variants. The most accurate method is wASTRID-s (tied with wASTRAL-h under the lowest level of missing data, the most accurate under higher levels). The ASTRID variants do not observably degrade in accuracy as the percentage of deleted taxa increases while the ASTRAL variants do. This relative robustness of weighted ASTRID to the clade-based missing data is largely expected given ASTRID’s robustness shown in the prior study of [47].

### Experiment 5: detailed comparison of the distance matrix algorithm

**Fig. 11:**
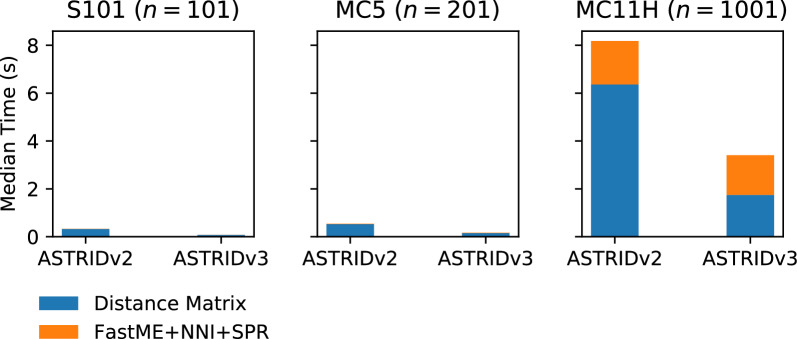
Wall-clock running times (in seconds) for ASTRID-2 and ASTRID-3, which differ only in the calculation of the distance matrix, on three different model conditions. Left: S101 with 101 species; middle: MC5 with 201 species; right: MC1H with 10001 species. Results shown are medians across 50 replicates. The portion in blue is for calculating the distance matrix, and the portion in orange is for running FastME

Recall that ASTRID variants first calculate an average distance matrix *D* from the gene trees, and then run FastME on *D*. From previously shown results in Table 2, wASTRID-s is clearly faster than ASTRID, only possible due to our change in the distance matrix calculation algorithm. To better benchmark this running time difference brought by this new algorithm for producing *D*, we broke down the running time of ASTRID-2 (the most efficient known implementation) and ASTRID-3, which is the ASTRID algorithm implemented inside our wASTRID software. We show the results in Fig. 11, benchmarking on 1000 gene trees.

The runtimes for both ASTRID-2 and ASTRID-3 on the two smaller datasets are both small, but with an advantage to ASTRID-3. However, on the largest dataset with 1001 species and 1000 genes, we see large differences in runtime. Unsurprisingly perhaps, the running time for FastME is essentially the same for both ASTRID and wASTRID, so that the runtime difference between the two methods is due to the time used to calculate the distance matrix, *D*. A detailed examination of that runtime (Table 3) shows that ASTRID-2 takes at least 3 times as long as ASTRID-3 to compute the distance matrix, across all conditions. The constant-time speed up is consistent with the fact that both ASTRID and wASTRID implement $O(n^2 k)$ algorithms for calculating *D*.

## Discussion

Here we discuss the trends seen in this study, and how they compare to trends from previous studies. We begin with a discussion about accuracy before turning to computational performance.

### Accuracy

#### Overall trends

For all model conditions, all tested methods achieved better accuracy when given more genes or as ILS or GTEE decreased. These trends are well known and quite expected for all summary methods [7]. The accuracy advantage of weighted methods over unweighted methods is present in nearly all conditions, but is larger for the conditions with shorter sequences (equivalently, the higher GTEE conditions) and fewer genes. This trend was already established for wASTRAL-h compared to ASTRAL in [25], but shows that wASTRID-s, just like ASTRAL, is able to better interpret the signal in the input through taking branch support into consideration.

In most cases, weighting improved accuracy for both ASTRAL and ASTRID, but not for the simulated mammalian dataset, where both ASTRAL and ASTRID had conditions where they were more accurate than their weighted versions, and not for the extreme missing data condition (80% taxa missing), where ASTRAL (but not ASTRID) had a condition where it was more accurate than its weighted version. While we do not understand why this occurs, it is noteworthy that both ASTRAL and ASTRID had conditions where they were more accurate than their weighted versions.

#### wASTRAL-h vs. wASTRID-s

ASTRAL and ASTRID are known to be among the most accurate summary methods under ILS, and their relative accuracy is dataset dependent, as also shown in our results. The relative accuracy of their weighted counterparts is clearly influenced by the accuracy of their base methods, as seen in the performance differences in the avian and mammalian simulation, where either wASTRID-s and wASTRAL-h can be the more accurate method. On the ASTRAL simulated datasets (ASTRAL-III S100, ASTRAL-II SimPhy), wASTRID-s and wASTRAL-h have comparable accuracy, with a small advantage to wASTRAL-h.

The improvement of wASTRAL-h over wASTRID-s may be due to its weighting also incorporating branch lengths. A branch-length weighted version of ASTRID (wASTRID-pl) has mixed accuracy compared to ASTRID, and does not compete against the support-weighted wASTRID-s in accuracy. In general, wASTRID-s and wASTRAL-h serve as accurate species tree inference methods under ILS and are more robust to GTEE than ASTRID and ASTRAL, two of the most accurate summary methods. Both can scale very well, with wASTRID-s much faster. However, the differences in accuracy are dataset dependent, just as the comparison between ASTRAL and ASTRID for accuracy seems dataset dependent.

#### Impact of missing data

In this study, we examined two models of missing data: the more commonly studied i.i.d. model and the $M_{\text {clade}}$ model. As expected, error rates increase with deletions of taxa, a trend that arises from reduction in information content (and has been noted in other studies [7, 47]). Of greater interest, however, is the relative impact on ASTRAL and ASTRID, and on their weighted versions. We see that ASTRAL (weighted and unweighted) is more accurate than ASTRID (weighted or unweighted) under the i.i.d. model of missing data, but the reverse occurs for the clade-based model of missing data; this trend was already observed in [47, 60] for ASTRID and ASTRAL, and it is noteworthy that it appears for their weighted versions as well.

We also see that weighted versions are generally more accurate than unweighted versions under missing data, except that ASTRAL is more accurate than wASTRAL-h for the condition of a very high degree of i.i.d. missing data. This trend was not reported in [25], but we note that they only examined very low levels of missing data (5% of the taxa deleted per gene). Thus, overall our findings are consistent with prior studies, and show both that the weighted versions of ASTRID and ASTRAL have comparable levels of robustness to missing data (with advantage to wASTRID for clade-based missing data and advantage to wASTRAL for i.i.d. missing data).

Given these trends, it is interesting to consider the statistical consistency guarantees for these methods under these models of missing data. Under the i.i.d. model, ASTRAL is statistically consistent while ASTRID is not [9, 47], but both methods are statistically consistent under the clade-based model. That said, the condition under which ASTRAL is consistent for the clade-based model requires that it be run in exact mode, rather than the heuristic mode where a set of allowed bipartitions is computed and the returned tree must draw its bipartitions from that set [47]. In this experiment, we explored ASTRAL only in heuristic mode (and hence not in such a way as to guarantee statistical consistency), and yet wASTRAL-h and ASTRAL both showed relatively good robustness, for both types of missing data. Similarly, it is worth noting that ASTRID is not statistically consistent under the i.i.d. model, and yet both ASTRID and wASTRID-s had accuracy that was fairly close to that of both ASTRAL and wASTRAL-h. Thus, all four methods (weighted and unweighted versions of ASTRAL and ASTRID) exhibited comparable robustness to missing data.

### Computational performance

All tested methods, with the exception of ASTRAL, exhibit good scalability for large datasets, as wASTRAL-h, wASTRID-s, and ASTRID can efficiently handle genome-scale data. For instance, wASTRAL-h, wASTRID-s, and ASTRID completed the analysis of the avian biological dataset, a genome-scale dataset, in under five minutes, while ASTRAL required more than a day [14]. On simulated conditions, ASTRAL was the only method that ever exceeded our computational constraints during experimentation. This may be due to ASTRAL’s quadratic dependency [56] on the number of allowed bipartitions for the constrained search, which includes all the bipartitions from the input gene trees. Given many gene trees and high ILS or high GTEE, this set of allowed bipartitions can be large, which can increase the runtime for ASTRAL.

While wASTRID-s and wASTRAL-h are both very scalable, wASTRID-s is clearly faster than wASTRAL-h. Despite wASTRAL-h’s improved algorithm and parallelism, wASTRID-s is still two orders of magnitude faster than wASTRAL-h, and hence might provide better scalability on large-scale data.

## Conclusions

While the estimation of species trees using summary methods, such as ASTRAL, ASTRID, MP-EST, and others, is now commonplace, it is known that gene tree estimation error reduces the accuracy of the estimated species tree. We presented support-weighted ASTRID (wASTRID-s), an improvement over ASTRID that incorporates uncertainty in gene tree branches into its estimation of the average intertaxon distance matrix. The development of wASTRID-s is inspired by the recent work of weighted ASTRAL (wASTRAL-h), which improved upon ASTRAL, and we showed that wASTRID-s obtained similar accuracy improvements over ASTRID. The advantage provided by wASTRID-s over ASTRID is most noteworthy under higher degrees of gene tree estimation error. wASTRID-s has very close accuracy to wASTRAL-h and is sometimes more accurate, but overall wASTRAL-h has a small advantage in accuracy, while wASTRID-s has an advantage in speed.

Although weighting usually helped accuracy for both ASTRID and ASTRAL, there were cases where it did not help, and even some cases where it reduced accuracy. All tested methods show a degree of robustness to missing data, but ASTRAL variants and ASTRID variants differ in their relative performance depending on the model of missing data.

This study was limited to datasets where the only cause for gene tree discordance with the species tree was ILS and gene tree estimation error. When considering real world datasets that may have other sources of gene tree heterogeneity, such as GDL or HGT, it seems likely that ASTRAL and other quartet-based methods may have an advantage over ASTRID and wASTRID-s, due to the theoretical proofs of statistical consistency for quartet-based methods for conditions involving gene duplication and loss or HGT (and the lack of such proofs for distance-based species tree estimation methods under the same conditions) [61–64].

Although wASTRID-s is highly accurate and very fast, we recommend using wASTRID-s in conjunction with wASTRAL-h and other species tree estimation methods. Due to its speed, the inclusion of wASTRID-s adds little computational burden, and having multiple different approaches for estimating the species tree, each based on a very different technique, can provide insights into what parts of the species tree are most reliably recovered, and which parts may need further data in order for full resolution.

This study suggests several directions for future work. Most importantly, finding a way to incorporate branch lengths into the branch certainty scoring for wASTRID-s, i.e. a hybrid weighting, could improve accuracy and might close the accuracy gap between wASTRID-s and wASTRAL-h under some conditions.

Another direction for future work is species tree estimation in the presence of gene duplication and loss, where gene family trees have multiple copies of species and so are called MUL-trees [65]. The combination of DISCO [66], a method for decomposing the MUL-trees into single-copy gene trees, with ASTRID produced very good accuracy and scalability [66], suggesting that combining DISCO with wASTRID might be even more accurate.

We also note that wASTRID might be useful for supertree estimation, a major challenge that currently has no fast and accurate methods that can scale to large datasets [67, 68]. Although ASTRID performed poorly as a supertree method for some model conditions [68], the reason for its poor accuracy may have been its reliance on the PhyD$^*$ methods for handling missing data (i.e., constructing trees from incomplete matrices). Given the more accurate handling of missing data introduced in this updated version of ASTRID and our introduced accuracy improvement taking branch support into account, wASTRID might prove to be a more accurate method for supertree estimation.

It may also be useful to use wASTRID to develop a weighted version of FASTRAL [11], a technique for speeding up ASTRAL. In FASTRAL, rather than building the set *X* of allowed bipartitions for the ASTRAL optimization criterion using the default setting (which can make *X* very large, as it includes by default all the bipartitions from all the input gene trees), the set of allowed bipartitions is taken from the species trees computed using ASTRID on random subsets of the gene trees. As shown in [11], FASTRAL maintains accuracy (and in some cases improves accuracy) compared to default ASTRAL, and can greatly reduce the runtime. Given the potential improvements in accuracy and the demonstrated improvement in speed, replacing ASTRID by wASTRID to produce a weighted version of FASTRAL might provide substantial improvements over FASTRAL.

Another direction for future work is to modify wASTRID to enable improved robustness to missing data, using (for example) the approach in Asteroid [60], which replaces the BME criterion by a revised criterion that better handles missing data.

This study explored accuracy using normalized Robinson-Foulds distances. Other studies [69], have suggested the use of alternative criteria, including quartet-similarity, to evaluate accuracy, when tree estimation is hampered by the presence of rogue taxa (i.e., taxa that can be added to a tree in many places). While these conditions did not arise in this study, which was largely based on simulated data, real-world datasets often do have such rogue taxa, and so subsequent studies should examine this question more carefully. As noted in particular in [69], this question may be particularly relevant for analyses of very large datasets, with more than 1000 species (the limit explored in this study). Hence, future work should also evaluate species tree estimation on biological datasets where rogue taxa are conjectured or known to create challenges, and use alternative accuracy criteria in those cases.

Although this study examined relatively large datasets (up to 1001 species and 1000 genes), larger datasets may create additional challenges.

For example, branch support in gene trees is known to be very low on large datsets, as discussed in [70], potentially making it difficult to reliably use weighted versions of summary methods, such as weighted ASTRAL and weighted ASTRID. Very large datasets also present computational challenges, and improving the speed for the distance matrix calculation through parallelization is a natural direction. Thus, future work should examine these questions in even larger phylogenomic datasets.

## Data Availability

Weighted ASTRID is available in open source form at [52]. All datasets, other than for Experiment 4, are from prior studies and freely available. For Experiment 4, we provide the modified gene trees, species trees, and scripts used to generate the data at [71]. The ASTRAL-III S100 and S200 data with aBayes support are available from weighted ASTRAL study at [72]. The “H”-suffixed data are available from [48]. The mammalian and avian simulated data are available from [73]. The avian biological data are available at [74].

## Appendix A: commands

### Weighted ASTRID

The commands differ depending on the type of support annotated on the gene trees. For FastTree SH-like, bootstrap support, and aBayes support, the commands for wASTRID-s are respectively:

$$\begin{aligned}{} & {} \texttt {wastrid -i \$genes -o \$output \# FastTree SH-like}\\{} & {} \quad \texttt {wastrid -x 100-i}{} \texttt { \$genes-o \$output \# bootstrap}\\{} & {} \quad \texttt {wastrid -n 0.333 -i \$genes -o \$output \# aBayes} \end{aligned}$$

For the final setting of wASTRID-pl (distance defined by path lengths, with branch lengths normalized by the maximum path-length in tree), the above commands need to be appended with an additional flag: -m n-length (distance using “normalized branch length” instead of the default -m support).

### Other summary methods

#### Weighted ASTRAL

We ran hybrid-weighted ASTRAL (v1.4.2.3) using the following command on trees annotated with aBayes support:

$$\begin{aligned} \texttt {astral-hybrid -x 1 -n 0.333 -i \$genes -o \$output} \end{aligned}$$

#### ASTRID

For all experiments except for Experiment 4, we ran ASTRID-2 compiled from scratch from [40] without missing data imputation, using the following command:

$$\begin{aligned} \texttt {ASTRID -s -i \$genes -o \$output} \end{aligned}$$

In Experiment 4, we instead used the following command:

$$\begin{aligned} \texttt {ASTRID -u -n -s -i \$genes -o \$output} \end{aligned}$$

#### ASTRAL

We ran ASTRAL (v5.7.8) using the following command:

$$\begin{aligned} \texttt {java -jar astral.5.7.8.jar -i \$genes -o \$output} \end{aligned}$$

#### FastME

ASTRID and wASTRID bind into and call subroutines inside FastME directly instead of calling FastME’s CLI interface. We bundle our software with FastME (v2.1.5). See https://github.com/RuneBlaze/internode/blob/3791b7e344869f3beb9f11e3d588e5e4eff788ba/src/internode.rs#L345 for how wASTRID binds into FastME, which is a translation of the C++ code from ASTRID.

### Obtaining gene tree branch support

#### Approximate Bayesian support

We computed IQ-TREE (v2.1.2) aBayes support using the following command:

where $aln is the alignment file for the gene tree, and $gtree is the path to the gene tree topology.

#### Bootstrap support

We computed bootstrap support (on the training dataset) using FastTree (v2.1.11) on bootstrap replicates generated by Goalign (v0.3.5) [75].

The following command was used to generate bootstrap replicates ($aln is the original gene alignment):

The FastTree command used for inferring a tree from one bootstrap replicate is ($bs_aln is one bootstrap alignment replicate generated by Goalign):

The FastTree bootstrap trees are then randomly resolved to eliminate polytomies, and then mapped by RAxML-NG [76] to the original gene trees using the following command:

where $gtree is the path to the original gene tree, and $bs_trees contains new-line separated newick trees inferred on the bootstrap replicates.

## Appendix B: additional text

### Failures to complete

ASTRAL failed to complete 24/50 replicates (replicates 2, 4, 6, 8, 9, 11, 13, 15, 16, 17, 20, 23, 27, 28, 29, 30, 32, 36, 38, 39, 40, 41, 48, and 49) on the MC11H condition ($n = 1001){\,under\,} k = 1000$. The 24 log files indicate that ASTRAL timed out on each of the replicates. An example of the last three lines of the log files is attached:

### Branch support

Many techniques are available to assess branch support. In the following paragraph, let $T$ denote the gene tree that we aim to annotate each non-leaf edge $e \in E(T)$ with support, and let $A$ be the gene alignment that we used to infer $T$.

The most common approach is the bootstrap method first proposed by Joe Felsenstein [51]. Briefly, a fixed number of “bootstrap alignments” $\{A_1, \ldots , A_k \}$ (kaparameter commonly set to 100 or 1000) are sampled with replacement from columns of A. Each bootstrap alignment $A_i$ has same number of columns as A. Boot straptrees $\{T_i\}$ are then infer red fromt. Variants of Felsenstein’s support exists, including Rapid Bootstrap Support [77] and Ultrafast Bootstrap [78] designed for speed. A different approach, the Transfer Bootstrap Expectation [69], was proposed in order to enable robustness to rogue taxa in large phylogenies.

This study extensively uses the approximate Bayes branch support [41], available in the IQ-TREE [49] software, which is an approximated version of the posterior probability of an NNI configuration at a particular branch. Similarly only exploring the NNI configurations around a branch, FastTree SH-like support, which relies on a Shimodaira-Hasegawa-like procedure [79, 80], was also explored in this study.

The Bayesian posterior probability support [81], a well known form of support approximating the posterior probability of the bipartition, seems computationally infeasible for datasets in this study $(n \ge 200)$ and is thought to be an overconfident form of support [82].

## Appendix C: additional table

**Table 3:** Wall-clock running times of ASTRID-2 and ASTRID-3, which differ only in the calculation of the distance matrix. Time are shown in seconds, median across 50 replicates

Running time (s)	Distance matrix		FastME	
	ASTRID-2	ASTRID-3	ASTRID-2	ASTRID-3
S101 (seqlen = 400, n = 101)	0.32	0.07	0.00	0.00
MC5 (n = 201)	0.53	0.15	0.01	0.01
MC11H (n = 1001)	6.37	1.75	1.82	1.66
